# Combined robot-assisted simple prostatectomy and laparoscopic nephrectomy: a case series

**DOI:** 10.1186/s13256-026-06039-2

**Published:** 2026-04-22

**Authors:** Narmina Khanmammadova, Bruce Gao, Johnny Wang, Dat Tien Nguyen, Daniel Jiang, Tuan Thanh Nguyen, Nick Hassas, Sohrab N. Ali, Kristene Myklak, Mohammed Shahait, David I. Lee

**Affiliations:** 1 Department of Urology, University of Califonia Irvine, Orange, CA USA; 2 Department of Urological Oncology Surgery, Binh Dan Hospital, Ho Chi Minh, Vietnam; 3 Division of Urology and Urologic Oncology, Department of Surgery, City of Hope, Duarte, CA USA; 4 Department of Urology, University of Medicine and Pharmacy, Ho Chi Minh, Vietnam; 5 Chicago Medical School, Rosalind Franklin University, North Chicago, USA; 6 Golden State Urology, Fremont, CA USA

**Keywords:** Robot-assisted simple prostatectomy, Benign prostatic hyperplasia, Laparoscopic, Nephrectomy, Renal cell carcinoma, Case series

## Abstract

**Background:**

The indications for robotic surgery in urology continue to expand with the evolution of surgical techniques and technologies. The feasibility of combined robotic/laparoscopic surgery for the treatment of synchronous upper and lower urinary tract malignancies has been previously described. However, to our knowledge, this is the first reported series of robotic-assisted simple prostatectomy (RASP) and robotic/laparoscopic nephrectomies performed in a single operative session.

**Case presentation:**

Case 1 involves an 80-year-old non-Hispanic White man of European descent with a history of low-risk prostate cancer who presented with lower urinary tract symptoms (LUTS) due to benign prostatic hyperplasia (BPH), and renal cell carcinoma (RCC). Robotic-assisted partial nephrectomy (RAPN) and RASP were performed sequentially, with modified port placements that allowed reuse of several trocar sites. Case 2 involves a 75-year-old North African man with a history of bilateral polycystic kidneys and stage IV chronic kidney disease who presented with LUTS due to BPH and unilateral RCC. This patient underwent RASP and conventional laparoscopic radical nephrectomy (LRN) in a single operative session. The total operative time was 221 min for Case 1 (94 min for RAPN and 77 min for RASP) and 255 min for Case 2 (104 min for LRN and 95 min for RASP). The estimated blood loss was 100 ml and 80 ml, respectively, with no transfusions required. Case 1 was discharged on post-operative day (POD) 1. Subsequent follow-up demonstrated alleviation of LUTS and no evidence of cancer recurrence. Case 2 was discharged on POD 3, with ongoing oncological surveillance.

**Conclusions:**

These cases demonstrate that combined RAPN or LRN with RASP can be performed safely even in patients with significant comorbidities. The success of these cases can be attributed to meticulous preoperative planning and involvement of a multidisciplinary care team. When feasible, combined surgery may offer benefits such as decreased risks associated with anesthesia and shorter hospitalizations.

## Background

Robotic surgery platforms are widely used across the various domains of minimally invasive urology, with nearly 85% of all radical prostatectomies in the United States performed robotically [1]. Robot-assisted approaches are also established for other major urologic procedures, including radical cystectomy and partial or radical nephrectomy [2]. As experience with robotic surgery grows, new combined procedures are also being explored, particularly in the management of synchronous upper and lower urinary tract malignancies.

The increased availability of cross-sectional imaging has led to more frequent discovery of incidental renal cell carcinoma (RCC) [3, 4]. For example, the incidental detection of synchronous primary tumors during prostate cancer (PCA) staging is approximately 1.5%, with RCC comprising a small percentage of these cases [5]. Several groups have demonstrated the feasibility of simultaneous robot-assisted partial nephrectomy (RAPN) and radical prostatectomy in select patients [6–13]. Here we present the pre-, peri-, and post-operative outcomes of two patients who underwent combined robot-assisted simple prostatectomy (RASP) with robotic/laparoscopic nephrectomy.

## Case presentation

### Case 1

An 80-year-old non-Hispanic White man of European descent with a history of small renal mass discovered on workup for gross hematuria presented with worsening LUTS secondary to BPH. His surgical history included prior colectomy for diverticulitis, transurethral resection of the prostate (TURP), and pacemaker implantation. His medical history was significant for a prior cerebrovascular accident.

**Fig. 1:**
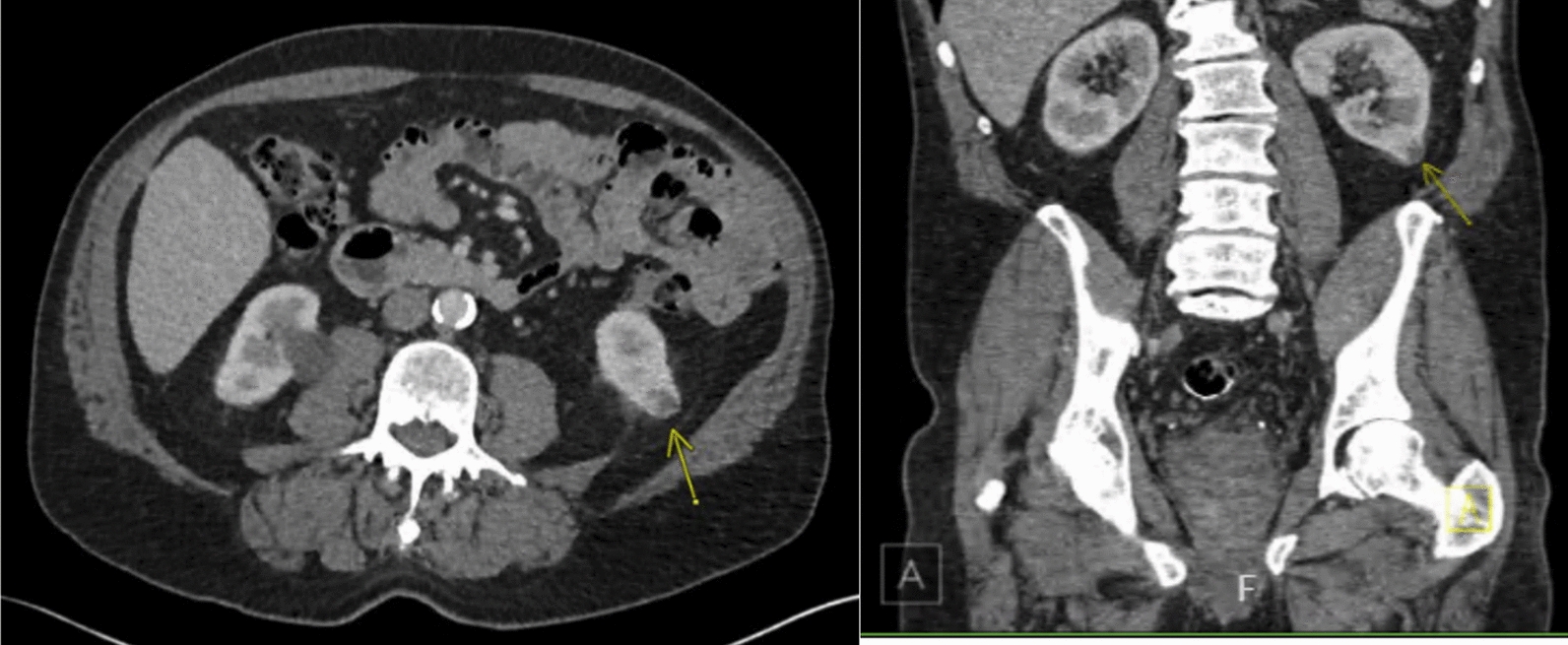
Case 1: Axial and coronal CT scan slices of biopsy-confirmed renal cell carcinoma

Contrast-enhanced computed tomography (CT) scan of the abdomen demonstrated a 1.61 × 1.75 × 2.08 cm exophytic renal lesion in the left lower pole (Fig. 1). Subsequent biopsy confirmed clear cell carcinoma, Fuhrman grade 2. The tumor was characterized as low complexity with a R.E.N.A.L. nephrometry score of 4p (R = 1, E = 1, N = 1, A = p, L = 1).

**Fig. 2:**
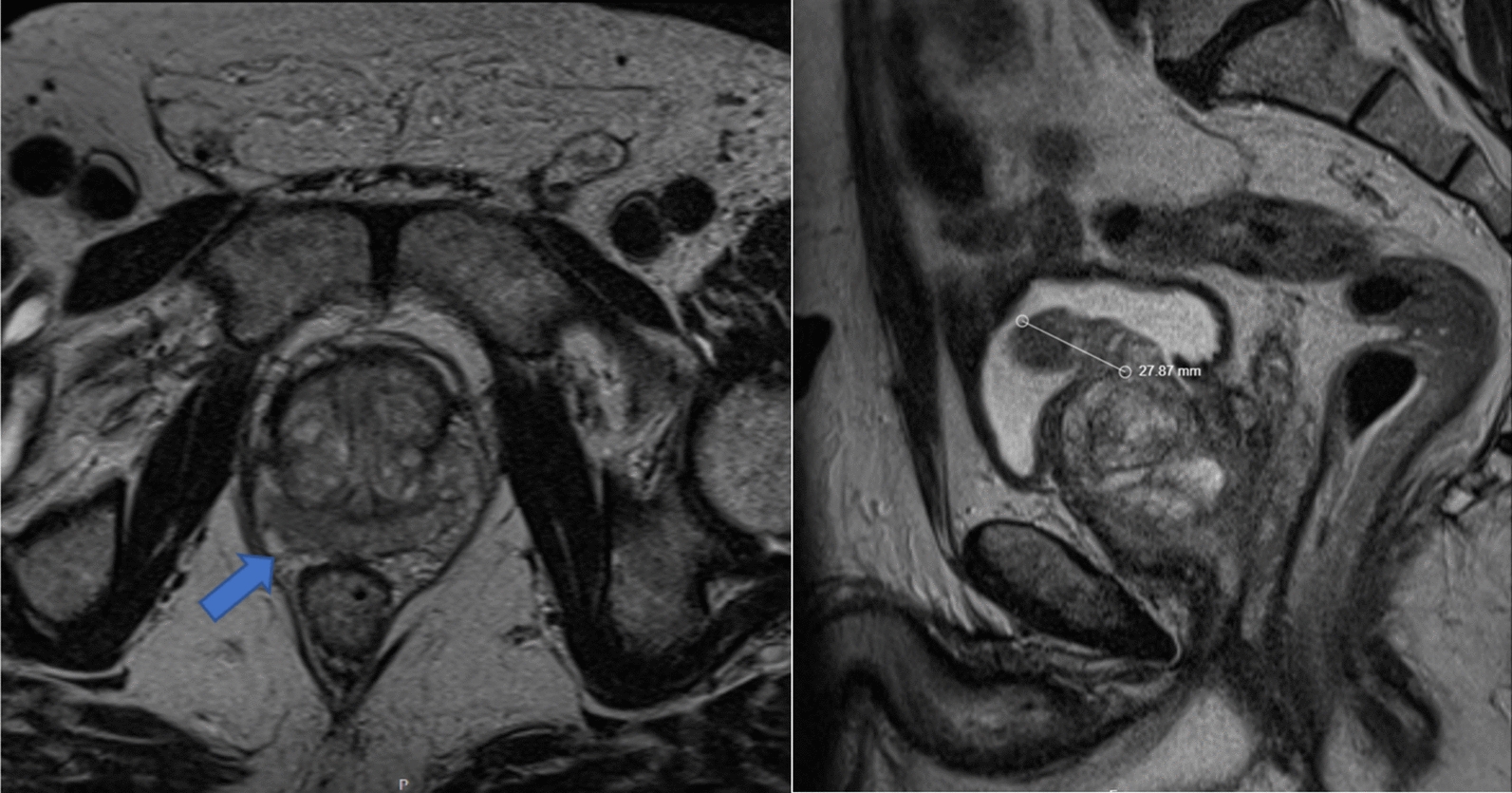
Case 1: Axial slice of multiparametric magnetic resonance imaging demonstrated a prostate imaging reporting and data system 4 lesion concerning for prostate cancer. Sagittal slice of multiparametric magnetic resonance imaging demonstrates an enlarged median lobe

During evaluation of his LUTS, the patient was found to have a serum total PSA of 4 ng/mL and serum creatinine of 1.01 mg/dL. Digital rectal examination at the time was unremarkable. Multiparametric magnetic resonance imaging (mpMRI) of the prostate revealed a PI-RADS 4 lesion in the right posterior peripheral mid-gland to apex, without extracapsular extension, seminal vesicle invasion, pelvic lymphadenopathy, or suspicious osseous lesions. The prostate volume was 136 ml with the presence of a substantial median lobe (Fig. 2). Prostate biopsy revealed low-grade, low-volume prostate adenocarcinoma with a Gleason score of 6 (ISUP grade 1) in 2 of 15 cores and a maximum core involvement of 10%.

Treatment options for RCC, PCA, and BPH were discussed with the patient. Given the patient’s age and comorbidity profile, the primary objectives were symptom relief and oncological control of the RCC, with a plan for active surveillance of the PCA. After deliberation, he elected for combined RAPN and RASP.

### Case 2

**Fig. 3:**
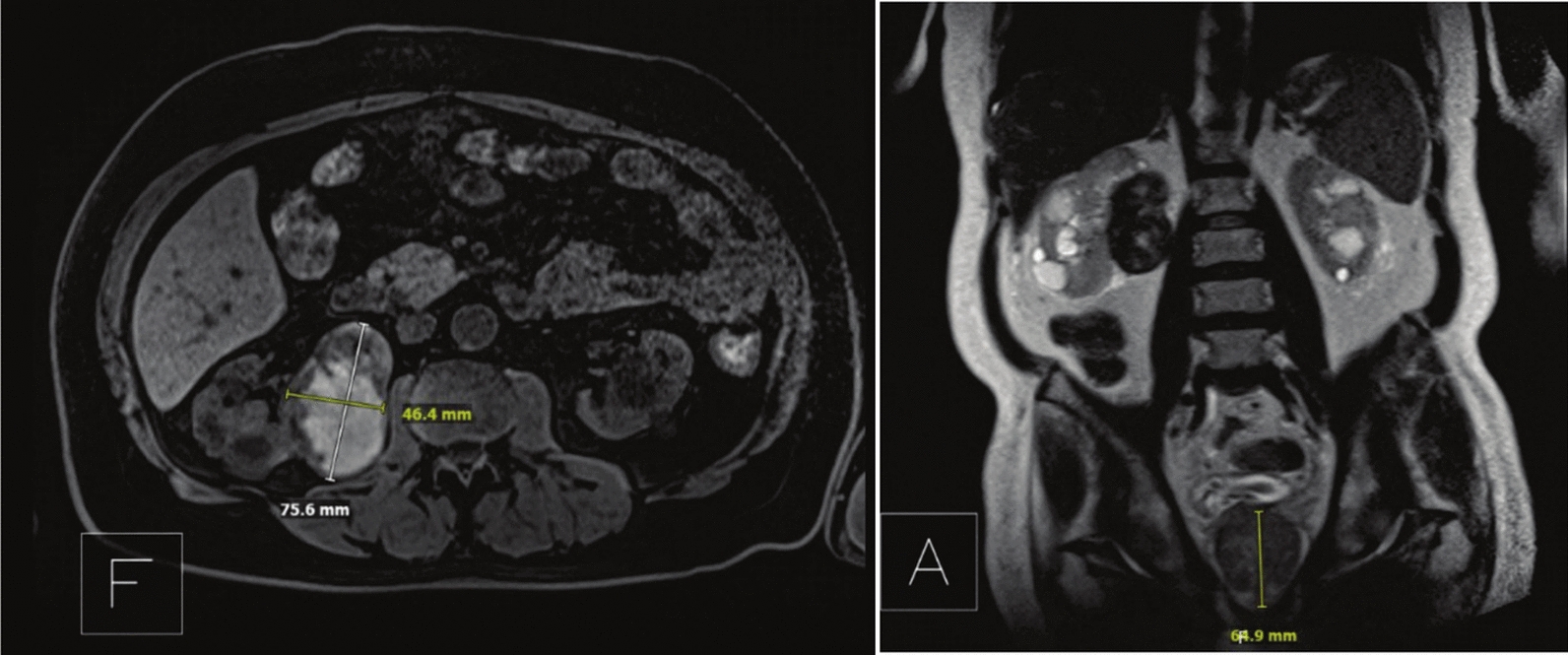
Case 2: Axial and coronal MRI slices demonstrated a large right renal mass and enlarged prostate

A 75-year-old North African man presented with a two-year history of worsening LUTS with American Urological Association Symptom Score (AUASS) of 10, refractory to medical therapy. His serum total PSA levels fluctuated between 7.2 and 17.6 ng/ml, and mpMRI revealed a markedly enlarged prostate (152.8 ml) without suspicious PI-RADS lesions (Fig. 3). Relevant medical history included polycystic kidney disease, stage IV CKD, hypertension, type 2 diabetes mellitus, gout, hyperlipidemia, and peripheral vascular disease. His BMI was 29.6 kg/m^2^, and he had a 50-pack-year smoking history. During evaluation for operative management of BPH, he developed new-onset right flank pain. Abdominal MRI and non-contrast CT demonstrated a 7.6 cm exophytic solid mass in the right interpolar region, with features highly suggestive of RCC (Fig. 3). Subsequent biopsy demonstrated ISUP grade 2 papillary type RCC. A R.E.N.A.L. nephrometry score of 10 × indicated high tumor complexity (R = 3, E = 1, N = 3, A = x, L = 4).

Owing to his underlying renal impairment, the patient received counseling with nephrology regarding the risk of progression to end-stage renal disease (ESRD) and postoperative hemodialysis with surgical management of his RCC. A multidisciplinary discussion was convened to address his LUTS and RCC. After reviewing risks and benefits, the patient elected for combined surgery. Preoperatively, the patient was evaluated by anesthesiology and planned for general endotracheal anesthesia with transversus abdominis plane block and arterial line for monitoring of fluid and electrolyte status. The procedure included RASP and right LRN with lymph node dissection (LND) due to intraoperative suspicion of reactive tumor tissue involving the paracaval lymph nodes, both performed in a single session. A hemodialysis catheter was placed in anticipation of postoperative renal replacement therapy.

### Case 1 surgical technique: off-clamp RAPN combined with RASP

**Fig. 4:**
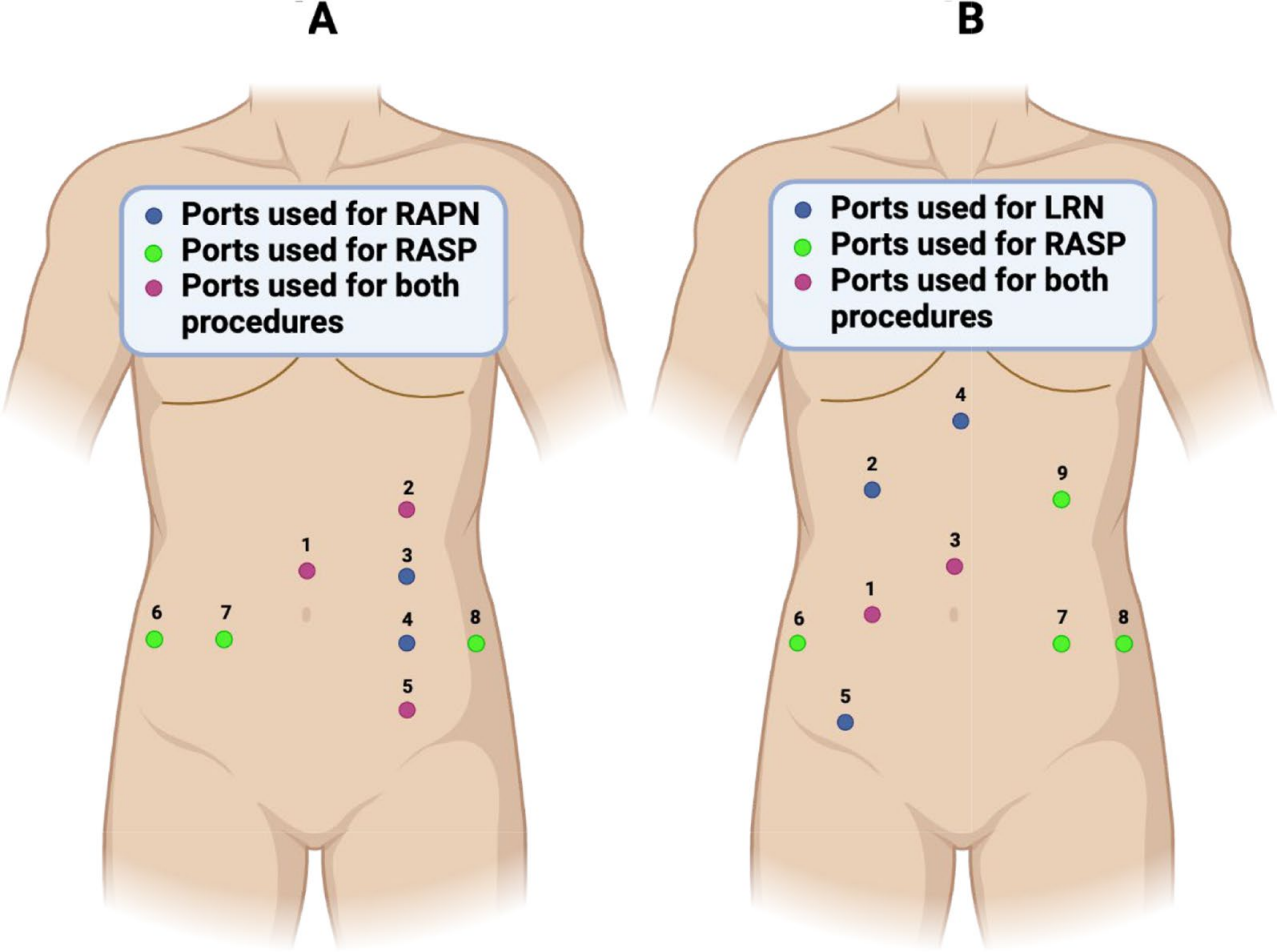
Diagram of trocar placement. Blue ports were used for robot-assisted partial nephrectomy/laparoscopic radical nephrectomy. Green ports were used for robotic-assisted simple prostatectomy. Purple ports were used for both procedures. Specimens were removed by extending the superior umbilical port incision. (Created in https://BioRender.com). **A** Diagram of trocar placement for concurrent robot-assisted partial nephrectomy and robotic-assisted simple prostatectomy. Port numbers: **1** → 12 mm AirSeal® assistant port (robot-assisted partial nephrectomy), 8 mm camera port (robotic-assisted simple prostatectomy); **2** → 8 mm robotic port (robot-assisted partial nephrectomy), 5 mm assistant port (robotic-assisted simple prostatectomy); **3** → 8 mm camera port; **4** → 8 mm robotic port; **5** → 8 mm robotic port (robot-assisted partial nephrectomy and robotic-assisted simple prostatectomy); **6, 7** → 8 mm robotic ports (robotic-assisted simple prostatectomy), **8** → 12 mm AirSeal® assistant port (robotic-assisted simple prostatectomy). **B** Diagram of trocar placement for concurrent laparoscopic radical nephrectomy and robotic-assisted simple prostatectomy. Port numbers: **1** → 12 mm left laparoscopic port (laparoscopic radical nephrectomy), 8 mm robotic port (robotic-assisted simple prostatectomy); **2** → 12 mm laparoscopic port (laparoscopic radical nephrectomy); **3** → 12 mm camera port (laparoscopic radical nephrectomy), 8 mm camera port (robotic-assisted simple prostatectomy); **4** → 5 mm laparoscopic port (liver retraction); **5** → 5 mm laparoscopic assistant port (laparoscopic radical nephrectomy); **6, 7** → 8 mm robotic ports (robotic-assisted simple prostatectomy); **8** → 12 mm AirSeal® assistant port (robotic-assisted simple prostatectomy) and **9** → 5 mm assistant port (robotic-assisted simple prostatectomy)

The daVinci Xi robotic platform (Intuitive Surgical, Sunnyvale, CA, USA) was used to perform both procedures. Both cases were performed by a team of two surgeons, with the same surgeon performing the nephrectomy across both cases and the same surgeon performing the RASP across both cases. The off-clamp RAPN was performed first to prioritize the vascular and oncologic portion of the case. The patient was placed in a right lateral decubitus position. After obtaining access to the abdominal cavity, insufflation pressure was initially set at 15 mmHg. Four 8-mm ports were placed in a row just lateral to the rectus muscle, and a 12-mm assistant port was placed at the midline just above the umbilicus (Fig. 4A). After successful port placement, insufflation pressure was reduced to 8 mmHg for the remainder of the case.

Following medialization of the colon, an intra-abdominal ultrasound was used to identify the tumor. Given its small size and exophytic nature, an off-clamp partial nephrectomy was performed. The resection base was oversewn with 3-0 V-Loc™ suture, and the renorrhaphy was completed with 0 Vicryl sutures and sliding clips. The specimen was placed in a retrieval bag intra-abdominally for subsequent removal.

For bladder neck sparing RASP, the patient was converted to supine Trendelenburg position. Prior to repositioning, two of the prior 8-mm ports were closed with 4-0 Biosyn™ suture and Dermabond™ skin adhesive, then covered in an Ioban™ antimicrobial drape. Subsequently, the patient was moved, re-prepped, and re-draped in the standard sterile fashion. The total turnover time between undocking the robot and incision time for the prostatectomy portion of the case was 40 min. A new 8-mm port was placed just above the umbilicus at the prior location of the 12 mm AirSeal port, through which the camera was introduced. The skin was partially closed with suture to prevent air leak. The remaining ports were placed under direct vision, with three 8-mm ports placed in a line just below the umbilicus, a 12-mm AirSeal port placed in the left lower quadrant, and a 5-mm assistant port in the left upper quadrant (Fig. 4A).

A transperitoneal approach was used to expose the prostatovesical junction. Our preferred method for RASP is to divide the bladder neck from the prostate in a similar fashion to radical prostatectomy. A plane around the adenoma was established and continued circumferentially and distally to the apex of the prostate. The anterior commissure was divided to expose the verumontanum, and the adenoma was removed. A 3-0 barbed suture was used to reapproximate the bladder neck and urethra in a bidirectional running fashion. The adenoma was placed in a retrieval bag and removed along with the renal mass by extending the periumbilical port incision. The kidney tumor was removed through the same incision. The periumbilical port site was closed in a running fashion with a 1 Maxon™ suture to ensure fascial closure. The remaining port sites were closed with 4-0 Biosyn™ suture and Dermabond™.

### Case 2 surgical technique: conventional LRN combined with RASP

As with the first case, LRN was performed first with the patient placed in the left lateral decubitus position. After establishing pneumoperitoneum, three 12 mm trocars were inserted: two along the anterior axillary line (one below the costal margin for the AirSeal, another inferiorly) and one medially between them, just lateral to the rectus. Two 5 mm trocars were placed under direct vision, one near the xiphoid process for liver retraction and one in the right lower quadrant for kidney retraction (Fig. 4B). Attention was then turned to incising the white line of Toldt, mobilizing the ascending colon, and Kocherizing the duodenum medially. The insertion of the gonadal vein on the inferior vena cava was identified, and a dissection plane superior to the gonadal vein was carried posterocaudally along the psoas muscle to the renal hilum. The retroperitoneal space was noted as inflamed and adherent around the hilum and tumor. The right renal artery and vein were identified, dissected, and transected using the Endo-GIA stapler. After sparing of the right adrenal gland and transection of the ureter, the right kidney, perinephric fat, and renal tumor were removed en bloc. A limited paracaval lymph node dissection was performed, given the reactive appearance of the tissue. The specimen was placed in an entrapment sac. The 12 mm trocar right lower quadrant primary access was extended by 6–7 cm in a modified Gibson incision to allow specimen extraction (Fig. 4B, port 1). This incision was closed in two layers.

In order for the second surgeon to perform the RASP portion of the case, the patient was returned to the supine position. The turnover, trocar placement, and operative steps were similar to the first case. However, the trocar positions were slightly modified (Fig. 4B), allowing for 2 trocars from the LRN portion of the case to be reused for RASP.

### Case 1 outcomes

Table 1 includes key patient characteristics and surgical outcomes for both cases. In the first case, the total operative time was 221 min. The console time for RAPN and RASP was 94 and 77 min, respectively. Estimated blood loss (EBL) was 100 cc, without the need for blood transfusion. After an uneventful postoperative course, the patient was discharged the next day after surgery. Creatinine remained at 1.4 mg/dL. Final pathology revealed 52 g of nodular prostatic hyperplasia negative for malignancy and clear cell RCC, WHO/ISUP grade 2, pathologic stage T1a with negative margins. At three-month follow-up, the patient endorsed a strong urinary stream with PSA of 0.83 ng/mL. Imaging showed no evidence of residual disease within the kidney.

**Table 1:** Characteristics of the patients and surgical outcomes

	Case 1	Case 2
Type of surgery	Left RAPN + RASP	Right LRN + RASP
R.E.N.A.L nephrometry score	4	10
Prostate size (cc)	136	152.8
BMI (kg/m^2^)	28.8	29.6
Time of surgery (minutes)		
Total operative time	221	255
RAPN/LRN	94	104
RASP	77	95
Number of ports used		
RASP only	3	4
RAPN/LRN only	2	3
Both	3	2
EBL (ml)	100	80
Day of discharge	POD1	POD3

### Case 2 outcomes

The total operative time was 255 min, including 104 min for LRN and 95 min of console time for RASP. The EBL was 80 ml. As anticipated, the patient’s GFR rose to 5.2 mg/dL by postoperative day (POD) 2. He was started on hemodialysis and discharged on POD 3. Final pathology revealed 64.3 g of benign prostatic tissue and papillary RCC type 1, ISUP grade 2, pT2aN0, with negative surgical margins. At two-week follow-up, the patient was recovering well and remained on hemodialysis. An MR urogram demonstrated no evidence of residual, recurrent, or metastatic disease. The patient discontinued BPH medication and reported significant improvement in LUTS (AUASS = 4) and quality of life (Quality of Life score = 0 / Delighted) at 6 months.

## Discussion and conclusions

The versatility of robotic platforms and experience of robotic surgeons have enabled increasingly complex procedures, including combined surgeries for synchronous upper and lower urinary tract disease [6–12, 14, 15]. Although prior reports of combined surgery have included robot-assisted radical prostatectomy for treatment of PCA, this series is among the first to highlight RASP. Potential benefits of a combined surgery include reduced hospital stay, convalescence time, risks associated with anesthesia, cost, and psychological stress [15]. Furthermore, reusing ports allows for lower trocar injury risk and better cosmetic results. Here, we report two combined upper and lower urinary tract cases for concurrent BPH and RCC. In the first case, eight total port site incisions were made; of the five port locations used for RAPN, three were reused for RASP. The 12-mm assistant port and most cephalad and caudal 8-mm RAPN ports were reused as the camera port, 5-mm assistant port, and 8-mm assistant port for RASP (Fig. 4A). Only small modifications to the standard port placements for RAPN and RASP were needed [16, 17]. Such port placements could help attenuate the learning curve for combined surgeries. In the second case, we reused two trocar positions from the LRN for the camera port and the second robotic arm during RASP.

There are some concerns about combined surgery, such as prolonged operation and anesthesia time, and risk of thromboembolism [18]. Despite prior recommendations to limit combined surgery to patients with minimal comorbidities, both of our cases involved patients with significant comorbidities, including stroke, arrhythmia, history of extensive abdominal surgery, hypertension, diabetes, and advanced CKD. After careful consideration of patient and tumor characteristics, a combined surgery was deemed feasible and cautiously attempted. Our total operative time was 221 and 255 min, comparable to the median operative time of 352 min (240–557) for combined upper and lower urinary tract surgeries reported by Scarcella et al. [15] In a recent study involving eight cases of RARP and RAPN, Drobot et al. reported median operative time, console time, and EBL of 315 min, 270 min, and 300 ml, respectively [19]. Although a simple comparison of operative times is inappropriate, a shorter operative time should be an important consideration for combined surgery in patients with comorbidities. Conversely, operative time is also an important consideration for surgeons. The favorable ergonomics of the daVinci platforms have enabled surgeons to take on longer procedures without significant fatigue.

Overall, the success of combined surgical procedures in frail patients can be attributed to meticulous preoperative planning and multidisciplinary collaboration. As an added benefit, we demonstrate that port sites can be reused with minor modifications to the standard configuration to reduce the total number of incisions. In select patients, combined surgery may offer benefits such as reduced hospitalization and recovery time, as well as decreased risks associated with repeated anesthesia inductions.

## Data Availability

The data used for the current study is available from the corresponding author on reasonable request.

## Abbreviations

PSA: Prostate-specific antigen
RCC: Renal cell carcinoma
PCA: Prostate cancer
RAPN: Robot-assisted partial nephrectomy
RASP: Robot-assisted simple prostatectomy
LRN: Laparoscopic radical nephrectomy
LUTS: Lower urinary tract symptoms
BPH: Benign prostatic hyperplasia
TURP: Transurethral resection of the prostate
CT: Computed tomography
BMI: Body Mass Index
AUASS: American Urological Association Symptom Score
mpMRI: Multiparametric Magnetic Resonance Imaging
PI-RADS: Prostate Imaging Reporting and Data System
ISUP: International Society of Urological Pathology
CKD: Chronic kidney disease
ESRD: End-stage renal disease
LND: Lymph node dissection
EBL: Estimated blood loss
POD: Postoperative day
GFR: Glomerular filtration rate
WHO: World Health Organization
RARP: Robot-Assisted Radical Prostatectomy
